# Planting the Seed for Blood Pressure Control: The Role of Plant-Based Nutrition in the Management of Hypertension

**DOI:** 10.1007/s11886-023-02008-z

**Published:** 2024-03-25

**Authors:** Justin A. Charles, Nilofer Khan Habibullah, Saul Bautista, Brenda Davis, Shivam Joshi, Sarah C. Hull

**Affiliations:** 1 Department of Family Medicine and Public Health, UC San Diego Health, San Diego, CA USA; 2 American International Medical University, Gros Islet, Saint Lucia; 3 Ethos Farm to Health/Ethos Primary Care, Long Valley, NJ USA; 4 Brenda Davis, Nutrition Consultations, Calgary, AB Canada; 5 Department of Veterans Affairs, Orlando, FL USA; 6 Department of Medicine, New York University Grossman School of Medicine, New York, NY USA; 7 Section of Cardiovascular Medicine, Yale School of Medicine, New Haven, CT USA; 8 Program for Biomedical Ethics, Yale School of Medicine, New Haven, CT USA

**Keywords:** Whole-food, plant-based diet, Lifestyle medicine, Dietary guidelines, Cardiovascular disease prevention, Dietary behavior change, Hypertension treatment

## Abstract

**Purpose of Review:**

Hypertension results in significant morbidity, mortality, and healthcare expenditures. Fortunately, it is largely preventable and treatable by implementing dietary interventions, though these remain underutilized. Here, we aim to explore the role of healthy dietary patterns in hypertension management and describe approaches for busy clinicians to address nutrition effectively and efficiently with patients.

**Recent Findings:**

DASH, Mediterranean, vegetarian, and vegan diets that include minimally processed, plant-based foods as core elements have consistently shown positive effects on hypertension*.* Recommendations that distill the most healthful components of these diets can significantly impact patient outcomes. Clinicians can harness evidence-based dietary assessment and counseling tools to implement and support behavioral changes, even during brief office visits.

**Summary:**

Healthful plant-based dietary patterns can often effectively prevent and treat hypertension. Clinicians may help improve patient outcomes by discussing evidence-based nutrition with their patients. Future work to promote infrastructural change that supports incorporating evidence-based nutrition into medical education, clinical care, and society at large can support these efforts.

**Supplementary Information:**

The online version contains supplementary material available at 10.1007/s11886-023-02008-z.

## Introduction

Hypertension (HTN) is the leading modifiable risk factor for premature cardiovascular disease (CVD), increasing the risk of coronary artery disease, arrhythmias, stroke, renal failure, and mortality [1–5]. It is increasingly common, affecting up to 45% (103–115 million) of US adults [6] and 31% (1.4 billion) of adults globally [7]. Even those who do not develop HTN by 45 have a lifetime risk of 80% to over 90% for developing HTN [8]. Only 40% of American adults with HTN are well-controlled, leaving tens of millions at risk [9]. HTN also accounts for $131 billion of healthcare costs, resulting in an additional $2000 of individual healthcare expenditure annually compared to those without HTN [10]. With increasing prevalence, complications, cost, and continued suboptimal control, further action to address HTN is imperative.

Guidelines from major medical organizations continue to recommend lifestyle modification, with diet as a primary component, as the first-line therapy for HTN [8, 11–13]. More specifically, minimally processed or whole-food, plant-based (WFPB) diets effectively prevent and treat HTN, along with many other cardiometabolic comorbidities [14]. Evidence-based nutrition interventions are under-taught in medical schools globally [15]. While physicians report insufficient training and knowledge regarding nutrition [16], patients view them as having nutrition expertise [17].

This paper aims to help address the gap between evidence-based therapeutic approaches, patient expectations, and clinician knowledge. It is not meant to be a complete literature review, as an in-depth review has been previously published [18••]. Rather, it should function as a primer for busy clinicians, providing clinically relevant information to improve the delivery of evidence-based, patient-centered dietary interventions to comprehensively address HTN.

First, we will review HTN-related clinical outcomes for WFPB diets and the proposed mechanistic evidence for their effectiveness. Then, we will describe dietary recommendations for patients with HTN and explain why and how clinicians should discuss nutrition with their patients, ways to overcome common barriers to adherence, and how to incorporate nutrition assessment and counseling into the workflow of busy clinical practice. Finally, we will explore future directions clinicians can take to promote a system that prioritizes ethical care of patients using the most effective treatment modalities.

While this paper focuses on nutrition, several other important lifestyle factors impact chronic disease, including HTN and CVD. The American College of Lifestyle Medicine (ACLM) describes six pillars of health. In addition to healthful eating, they include physical activity, restorative sleep, stress management, social connection, and avoiding the use of harmful substances [19].

## Evidence-Based Dietary Patterns

Various dietary patterns have been described to reduce systolic blood pressure (SBP) and diastolic blood pressure (DBP). Current recommendations by the American Heart Association (AHA) include an eating pattern that emphasizes fruits, vegetables, whole grains, predominantly plant-based proteins (legumes and nuts), fish, and seafood [13]. For those who choose to still consume meat or poultry, they advise selecting lean cuts and avoiding processed forms. Recommendations also include avoiding ultra-processed foods, which contain added salt, sugars, or fats, and artificial colors, flavorings, and preservatives.

Several dietary patterns have been evaluated for their consistency with AHA dietary guidance. The DASH, Mediterranean, pescetarian, and vegetarian (ovo, lacto, ovo/lacto) have been characterized as tier 1 dietary patterns, those with scores > 85 for alignment with the 2021 AHA Dietary Guidance [20•]. Tier 2 patterns score from 75 to 85 for alignment with 2021 AHA Dietary Guidance and include vegan and low-fat diets [20•]*.* Recent systematic reviews note that plant-based dietary patterns result in superior HTN, and cardiovascular-related health outcomes compared to animal-based diets [21, 22]. As this paper will outline, optimizing the quality of plant-based dietary patterns has the potential to immensely impact the prevention and treatment of HTN [23].

### DASH Diet

The Dietary Approach to Stop Hypertension (DASH) diet is one of the most effective dietary patterns for preventing and treating HTN [24, 25]. It emphasizes fruits, vegetables, and low-fat dairy foods; includes whole grains, poultry, fish, and nuts; and minimizes fats, red meat, sweets, added sodium, and sugar-containing beverages [26]. Interestingly, the initial premise of the DASH diet was to “have the blood pressure-lowering benefits of a vegetarian diet, yet contain enough animal products to make it palatable to nonvegetarians,” after earlier studies showed those consuming vegetarian diets had the lowest blood pressures in industrialized societies [27].

Recent systematic reviews and meta-analyses demonstrate the impact of DASH diets on the prevention and treatment of HTN [28, 29]. Those with the highest, compared to the lowest, adherence to the DASH diet resulted in a 20% decreased risk of developing HTN [28]. Additionally, compared to a control diet, the DASH diet significantly reduces SBP by 3.2 mm Hg and DBP by 2.5 mm Hg regardless of HTN diagnosis [29]. Individual clinical trials are even more impressive, with mean SBP reductions from 5.3 to 20.8 mm Hg for those with SBP < 130 and ≥ 150 mm Hg, respectively [30].

### Mediterranean Diet

A Mediterranean diet (MedDiet) represents commonalities of diets in Mediterranean countries [31]. It consists of high consumption (every meal) of fruits, vegetables, whole grains, nuts, and legumes; moderate consumption (daily to weekly) of fish, poultry, and dairy products; limited intake (less than twice weekly) of red meat and sweets; and red wine “in moderation” [32]. The MedDiet also emphasizes using unsaturated fats, mainly olive oil, as the primary source of added fat. Two recent meta-analyses exploring the relationship between MedDiet and HTN specifically noted small but significant average reductions in SBP (1.44–1.5 mm Hg) and DBP (0.7–0.9 mm Hg), with greater reductions achieved in those with higher baseline SBP [33, 34].

Further analysis of the MedDiet shows its main benefit is from low meat consumption and high intake of vegetables, fruits, nuts, and legumes, with no additional benefit from the consumption of fish and dairy [35]. Additionally, while the Mediterranean diet includes moderate wine consumption, recent evidence suggests that no amount of alcohol can be deemed unequivocally beneficial for HTN and overall health [36]. We note that the healthful effects traditionally attributed to wine may instead reflect those of other lifestyle factors, such as social connection.

### Vegetarian and Vegan Diets

Lacto-ovo vegetarian diets exclude all animal flesh—meat, game, poultry, fish, and shellfish—but include dairy and eggs, while vegan diets exclude all animal products. Large cross-sectional studies have demonstrated an inverse relationship between the restriction of animal products and both age-adjusted HTN prevalence and blood pressure readings, with vegans having the lowest, omnivores having the highest, and vegetarians and pescatarians in between [37–39]. Meta-analysis data demonstrated that vegetarian dietary patterns reduced SBP by 4.8–6.9 mm Hg and DBP by 2.2–4.7 mm Hg compared to omnivorous diets [40]. More recent meta-analysis data suggest that while non-calorically restricted vegan diets do not outperform non-vegan diets in all-comers, they do among those with elevated SBP above 130 mm Hg, resulting in an average decrease in SBP and DBP of 4.10 mm Hg and 4.01 mm Hg, respectively [41].

While more healthful plant-based diets decrease HTN risk, vegan and vegetarian diets in which unhealthy, highly processed plant-based foods predominate can increase the risk of HTN [42]. This highlights the importance of not only limiting animal-based dietary components but also ensuring the high nutritional quality of included plant foods.

### Whole-Food, Plant-Based (WFPB) Diet

The dietary patterns above are associated with beneficial outcomes, largely due to their common factors, including minimally processed, plant-based foods while limiting processed foods and animal products. A whole-food, plant-based (WFPB) diet is a rigorous variation on these shared healthful components, maximizing consumption of minimally processed, plant-based foods and minimizing or excluding all processed foods and animal products, including red meat, processed meat, poultry, fish, eggs, and dairy products [43]. The individual whole, plant-based food groups—fruits and vegetables [44], legumes [45], whole grains [46], nuts and seeds [47], and herbs and spices [48]—have each been shown to improve HTN-related outcomes.

## Mechanistic Evidence

A balanced WFPB diet exerts its blood-pressure-lowering effect through several mechanisms, including promoting weight loss, minimizing sodium, containing adequate levels of health-promoting micronutrients and phytonutrients, and avoiding harmful components of animal foods [18••].

### Weight Loss

Higher BMI is linked to a higher incidence of HTN, and weight loss often improves blood pressure [49, 50]. In fact, in some studies, BMI accounts for up to 50% of the blood pressure variations between diet groups [37]. WFPB diets have been shown to effectively promote weight loss and improve HTN [51].

### Sodium and Potassium

Sodium consumption is a significant risk factor for HTN [52], responsible for up to 9–17% of the population attributable risk [53]. Sodium-restricted diets, such as the DASH and WFPB diets, stress the avoidance of highly processed foods, the predominant source of dietary sodium in the USA [54], and have been shown to decrease blood pressure in large meta-analyses [55].

Potassium has been shown to reduce blood pressure by improving vasodilation, reducing vascular tension, and promoting natriuresis [56–58]. The most concentrated sources of potassium include legumes, fruits, and starchy and non-starchy vegetables.

The sodium-potassium-ratio is a stronger risk factor for HTN, CVD, and mortality than either element alone [59–61]. As most Americans both overconsume sodium [62] and underconsume potassium [63], dietary patterns for patients with HTN should aim to correct this imbalance.

### Magnesium and Calcium

Magnesium intake is inversely related to blood pressure and can help prevent and treat HTN by promoting healthy endothelial function and vasodilation [64, 65]. Magnesium is concentrated in leafy greens, legumes, seeds, nuts, whole grains, and other high-fiber foods.

While low calcium intake promotes vasoconstriction and increased peripheral vascular resistance [66, 67], sufficient intake appears beneficial for preventing and treating HTN [68, 69]. Though often associated with dairy in Western cultures, there are many excellent plant sources of calcium, including low-oxalate green leafy vegetables, tofu, legumes, nuts, seeds, and nondairy milks.

### Fiber, Naturally Occurring Nitrates and Phytonutrients

Only 5% of US adults meet daily recommendations for fiber [70]. Fiber reduces the risk of HTN and has been shown to reduce BP independently of its effect on weight loss in a meta-analysis of clinical trials [71, 72]. High-fiber plant-based diets may also beneficially affect the gut microbiome composition [73], which may help regulate blood pressure [74]. Dietary fiber is plentiful in whole plant foods but is not naturally present in animal products.

Nitrate-rich leafy green vegetables reduce inflammation and enhance nitric oxide production, which relaxes vascular smooth muscle and thereby lowers blood pressure [75, 76]. Beetroot juice has also been shown to reduce blood pressure among patients with HTN in several systematic reviews through nitrate-dependent and independent mechanisms [77–79]. However, due to high oxalate content, significant beet consumption may not be advisable for patients prone to nephrolithiasis [80]. In contrast to naturally occurring nitrates, synthetic nitrates and nitrites used to preserve processed meats are linked to the production of nitrosamines, which are known carcinogens and may also contribute to higher blood pressure [81, 82].

Phytonutrients, such as polyphenols and plant sterols, can help to control HTN via antioxidant, anti-inflammatory, vasodilatory, and apoptosis-inducing pathways [83–85]. The most concentrated sources are colorful whole-plant foods such as vegetables, fruits, legumes, whole grains, nuts, seeds, herbs, spices, and teas.

### Animal Food Components

Diets higher in animal foods tend to be associated with a greater risk of HTN [38, 39, 86–88]. They contain higher levels of saturated fat [89, 90] and advanced glycation end products (AGEs) [91] and promote the formation of trimethylamine-N-oxide (TMAO) [92], which have all been linked to HTN and CVD. A more comprehensive review of the health harms of animal food consumption can be found elsewhere [93•].

## Dietary Recommendations for HTN

**Fig.1:**
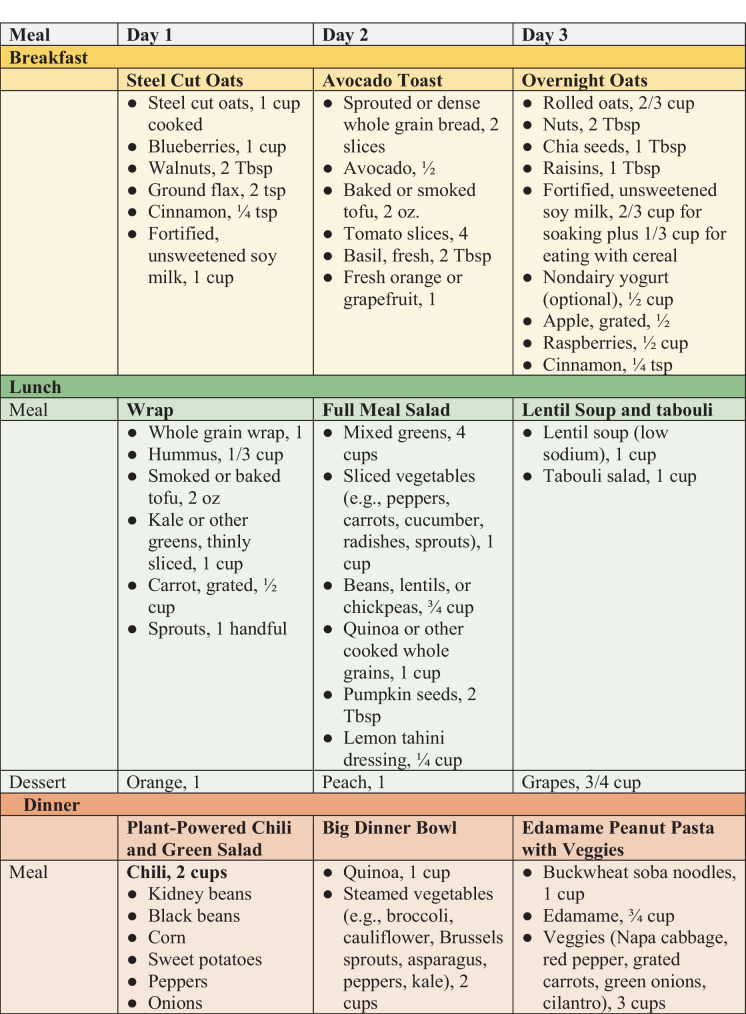

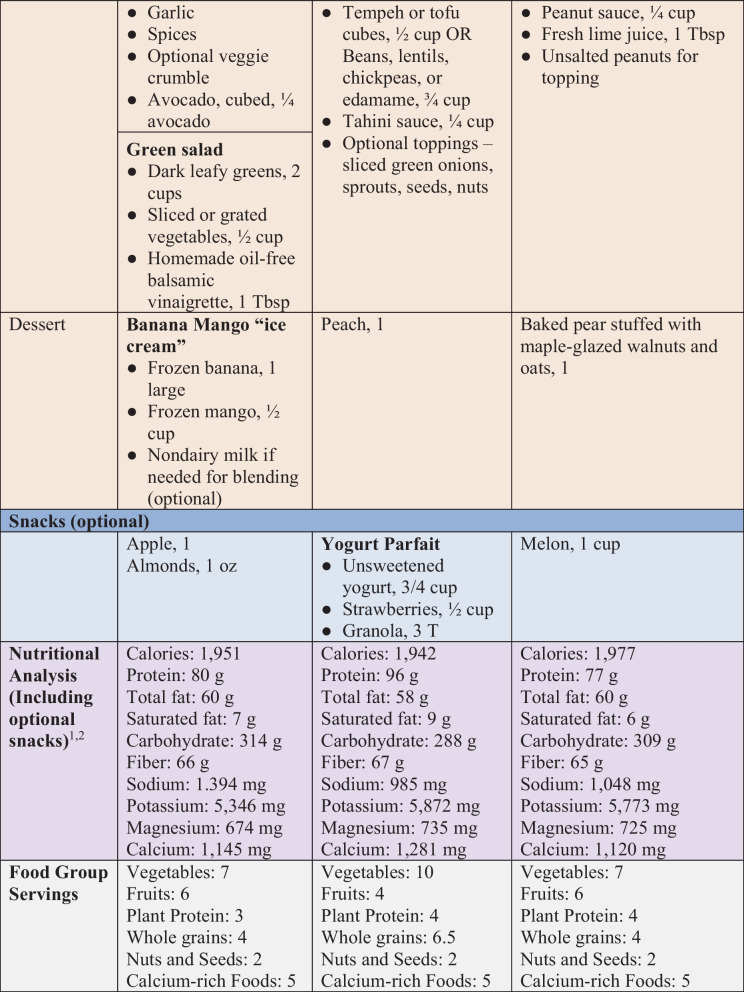
Sample menus for treatment of hypertension. ^1^All menus are under 2000 cal. Fiber ranges from 66 to 75 g. Protein ranges from 80 to 96 g. Saturated fat ranges from 3 to 4% of total calories. Sodium averages less than 1200 mg over 3 days. Potassium is over 5000 mg, magnesium is over 600 mg, and calcium is over 1100 mg. These levels all meet the recommendations outlined above. The only nutrition shortfalls are for vitamin B12, vitamin D, and iodine, for which supplementation is recommended. ^2^Menu was created using ESHA Food Processor Nutrition Analysis Software [95] and USDA FoodData Central [96]

While consuming adequate amounts of specific key nutrients is important for individuals with HTN, the most effective way to address HTN is with an overall health-promoting dietary pattern, as in Table 1. These recommendations are illustrated in a 3-day sample menu, as shown in Fig. 1. The foods included are healthy sources of macronutrients and rich sources of dietary fiber and micronutrients that promote vascular health. Additionally, though beyond the scope of this article, such plant-based dietary patterns can powerfully affect cardiovascular and metabolic health beyond HTN alone [94].

**Table 1:** Dietary guidelines for treating hypertension with WFPB diets

1. **Make the foundation of the diet whole plant foods** Include the following foods each day: • ***5 or more servings of vegetables*** ○ One serving = 1 cup raw leafy greens or ½ cup cooked or raw vegetables ○ Choose mostly dark, leafy greens and colorful non-starchy vegetables • ***4 or more servings of fruits*** ○ One serving = 1 medium-sized fruit or ½ cup fresh, frozen, or unsweetened canned fruit ○ Include berries, citrus fruits, and a variety of other fruits • ***3 or more servings of whole grains*** ○ One serving ■ ½ cup cooked grains ■ 1 slice whole grain bread ○ Vary intake according to calorie needs ○ Select mostly intact grains, such as barley or quinoa, and minimally processed grains such as steel cut or rolled oats • ***3 or more servings of plant-based proteins*** ○ One serving ■ ½ cup beans, lentils, split peas, tofu, tempeh, or seitan ■ 1 cup raw peas or sprouted lentils or peas ■ ¼ cup peanuts ■ 2 Tbsp peanut butter ■ 2 oz vegetarian meat substitute ○ Select mostly unprocessed legumes or lightly processed products such as tofu, tempeh, or seitan ■ If meat substitutes are used, select whole food-based options and compare sodium content • ***1 or more servings of nuts or seeds*** ○ One serving ■ 1-oz nuts or seeds ■ 2 Tbsp nut or seed butter (including peanut butter) ○ Select those without added salt, oil, or sugar • ***5 or more servings of calcium-rich choices*** ○ One serving ■ ½ cup fortified nondairy milk or yogurt ■ 2 cups raw or 1 cup cooked low-oxalate greens (e.g., broccoli, Bok choy, kale, mustard greens, Napa cabbage, turnip greens) ■ 1 cup soybeans, white beans, or black beans ■ ½ cup calcium-set tofu ■ ¼ cup almonds or 2 Tbsp almond butter ■ 2½ Tbsp chia seeds • ***Generous amounts of herbs and spices*** ○ Add herbs and spices to most meals ○ Good choices include basil, black cumin, cardamom, cayenne, celery seed, cinnamon, garlic, ginger, oregano, parsley, thyme, and turmeric
2. **Ensure sufficient consumption of dietary fiber** • Aim for a daily fiber intake of 14 g per 1000 cal ■ For age 18–50 y, 25 g for women and 38 g for men ■ For age 51 y and older, 21 g for women and 30 g for men • Include a wide variety of fiber-containing foods, including those rich in soluble fiber such as legumes, oats, barley, flaxseeds, and chia seeds
3. **Limit sodium to < 1500 mg per day** To reduce sodium intake: • Minimize processed foods which are the largest sources of dietary sodium • Compare food labels for canned and packaged food and choose no added salt or low salt options • Minimize added salt in cooking by using sodium-free salt substitutes (e.g., potassium chloride), herbs and spices, or acid-containing foods like citrus fruits or tomatoes • Leave the saltshaker off the table • Make your own sauces and dressings • Eat less restaurant food. Eat at home
4. **Minimize intake of added sugars** • Aim for no more than 5% of calories as added sugars or 6 teaspoons of sugar in a 2000-cal diet (1 tsp = 4 g on food labels) • Avoid sugar-sweetened beverages • Limit intake of sugar-sweetened treats
5. **Minimize added fats** • Avoid solid fats such as butter, margarine, shortening, and tropical oils • If oils are used, keep portions as small as possible • Select less processed options such as extra-virgin olive oil, avocado oil, and omega-3-rich oils • Avoid cooking with omega-3-rich oils as they readily oxidize at higher temperatures
6. **Include reliable sources of omega-3 fatty acids** • Sources of ALA include flaxseeds, chia seeds, hemp seeds, and walnuts • Consider including direct sources of EPA/DHA such as microalgae supplements • For those who include fish, select omega-3-rich choices that are lower in mercury, such as salmon, whitefish, trout, mackerel, and sardines
7. **Make plants your primary protein sources** • Minimize intake of animal protein sources • Avoid red and processed meats and limit whole egg or egg yolk consumption • If animal protein sources are consumed, select fish, lean poultry, and egg whites
8. **Include rich sources of antioxidants and anti-inflammatory foods at each meal** The most concentrated sources are whole plant foods such as: • Leafy greens and other colorful vegetables • Fruits, especially berries • Legumes, especially colorful red or black choices • Whole grains, especially colorful red, purple, or black choices • Nuts and seeds • Sprouts • Fermented foods • Herbs and spices • Green and herbal teas
9. **Avoid highly processed foods** • Minimize intake of refined starches such as white flour breads, and other baked goods, crackers, and fried or salty snacks
10. **Ensure nutritional adequacy** • Dietary Reference Intakes (DRIs) of key nutrients for adults with HTN ○ Potassium: 3400 mg for men, 2600 mg for women ○ Magnesium: 420–430 mg for men, 310–320 mg for women ○ Calcium: 1000 mg (1200 mg for women over 50 y and men over 70 y) • For those eating plant-based diets, include reliable sources of vitamin B12, vitamin D, and iodine, and. Use appropriate supplements as warranted

## Implementation

While identifying specific dietary recommendations and the evidence behind them is important, their utility is determined by clinicians’ ability to integrate them into clinical care. While a detailed review of dietary assessments, counseling techniques, and nutrition prescriptions is beyond the scope of this paper, this section will provide a brief overview of them along with existing comprehensive references for those who wish to learn more.

### Approaching the Conversation

It is important not to justify withholding minimal counseling by assuming patients are uninterested, unwilling, or unable to make lifestyle changes. This not only compromises beneficence but also threatens patient autonomy by failing to provide transparent information about the risks and benefits of all potential treatment options [97]. Learning that dietary interventions can lead to comparable or even more profound benefits than medication with less risk of side effects may sway patients to consider lifestyle changes. While the prospect of avoiding or deprescribing antihypertensives may be a powerful motivator, it is important to manage both patient and clinician expectations. Some patients will not make significant lifestyle changes, and others may still require pharmacotherapy despite adherence to dietary recommendations. Therefore, the clinician should balance communicating with patients in line with their readiness and willingness to change while clearly outlining optimal treatment goals. A reasonable first step clinicians can take is initiating a conversation about nutrition with patients [98].

### Dietary Assessment

Several rapid dietary screening tools can accurately assess patients’ nutritional status. Vadiveloo et al. [99] present a flowchart with recommendations for busy clinicians. They recommend using the *Nutrition Screening Protocol Questions* [100] when clinicians have less than 5 min, *Starting the Conversation* tool [101] for visits between 5 and 10 min, and the *Mediterranean Diet Adherence Screener* [102] for visits longer than 10 min. For those with more time, it can be helpful to perform a 24-h dietary recall [103].

### Counseling Basics

In-office counseling using evidence-based techniques, such as the *5 A*s model, brief action planning (BAP), and motivational interviewing (MI) can help with goal setting and behavior change in busy clinical settings. Importantly, they involve coaching patients to set their own goals, which is more likely to improve self-efficacy and result in behavioral change than dictating goals using an expert approach [104].

The *5 As* model (ask, assess, advise, agree, assist) is a theory-based, patient-centered, practical framework for discussing obesity and dietary behaviors [105]. Brief action planning (BAP) is a self-management support technique used to assist an individual in creating an achievable action plan for health behavior change [106]. With practice, BAP can be conducted with patients in less than 5 min. Motivational interviewing (MI) is a patient-centered communication style that aims to resolve ambivalence to behavior change by identifying and encouraging patients’ internal motivation and commitment to change [107]. Using MI has been shown to positively impact HTN outcomes [108–110]. A recent excellent editorial outlines the key principles and necessary steps to incorporate BAP and MI into patient care [111].

### SMART Goals and Nutrition Prescriptions

SMART goals are specific, measurable, achievable/attainable, relevant/realistic, and time-sensitive. Accompanied by step-by-step action plans, they define the what, where, when, and how of goal-directed behavior change [112]. Nutrition prescriptions apply these concepts to provide specific, time-bound dietary recommendations to patients about the type, amount, and frequency of food that should be consumed or avoided [113]. Similarly to pharmacologic prescriptions, it is essential to prescribe the right “dose” or intensity of intervention to help patients meet their goals and produce desired clinical outcomes. A further explanation of SMART goal-informed nutrition prescriptions with examples can be found in supplementary Table S1. Supplementary Table S2 demonstrates the application of the 5*A*s model, BAP, and MI techniques to develop a sample nutrition prescription for patients with HTN.

### Addressing Barriers to Change

Barriers to dietary health behavior change can range from the individual level to the complex interplay between interpersonal, organizational, environmental, and public policy factors [114]. This review focuses on the individual level, which has greater potential to be addressed within a traditional clinical encounter. In her 2008 article, Robinson outlines that individual barriers to improving dietary behavior “include taste preferences, lack of knowledge and/or belief in the association between diet and health, habits, and self-efficacy [115]”. These can generally be divided into insufficient knowledge, motivation, or skills needed to execute dietary change. Lack of knowledge can be addressed by providing patients with evidence-based resources outlining basic information, success strategies, recipes, and sample meal plans. Clinicians can address difficulty with initiating behavior by identifying and discussing barriers to behavior change, sharing resources to learn cooking skills, and referring to registered dietitians or health coaches as appropriate. To address issues with motivation, clinicians can use motivational interviewing to determine the cause of the lack of motivation, develop discrepancies between the current behavior and the desired outcome, provide resources for social support, and refer to behavioral health specialists, social workers, or health coaches to help navigate challenges. Karlsen and Pollard outline several additional strategies to address patient concerns about plant-based nutrition and increase engagement [116]. Supplementary Table S3 outlines recommended resources clinicians can share with patients to help them overcome these barriers, such as dietary guides, cookbooks, and therapeutic lifestyle change programs.

Clinicians also experience barriers to addressing lifestyle changes during visits, namely the difficulty of changing patient behavior, lack of sufficient time, and issues with patient adherence [117]. We hope the strategies and resources referenced in this article can help overcome those barriers. However, this is sometimes outside of the clinician’s expertise and scope of the practice, and it may be more appropriate to refer to allied health professionals, such as health coaches, dieticians, or behavioral specialists, to assist the patient.

### When to Refer to a Registered Dietitian or Health Coach

Providing in-depth, individualized nutrition advice is outside the scope of practice of most physicians, who do not typically have the time or expertise to offer these consults [118]. However, these skills are within the scope of registered dietitians (RDs), health and wellness coaches, and other lifestyle support staff.

RDs have the training to teach patients how to implement the diet their physician recommends in a culturally appropriate, acceptable, and effective manner. They also have the time and ability to conduct more intensive nutritional assessments and address dietary concerns among patients longitudinally. For this reason, patients who receive dietary prescriptions, who have comorbidities, such as eating disorders, type 2 diabetes, or cardiovascular disease, or who have undergone bariatric surgery should be referred to RDs. Studies demonstrate better target outcomes when RDs are included in team-based care [119].

Health and wellness coaches can also be key players in helping patients with nutritional habits. They offer a patient-centered approach, using goal setting, identifying and overcoming barriers, and accountability to help patients navigate the day-to-day challenges of behavior change [104]. Data shows that health coaching effectively promotes healthy lifestyle behaviors, such as diet, and improves outcomes among patients with chronic diseases, including HTN [120, 121].

Group classes or support groups led by RDs and health coaches provide an opportunity to share ideas, knowledge, challenges, and successes and offer ongoing social support integral to positive, sustainable behavior change [122]. Ultimately, collaboration between nutrition professionals and the medical team can ensure that patients receive the support they need to achieve their diet and lifestyle goals.

### Visit Logistics

Behavior change takes time; not everything can be covered in one visit by one clinician. Initial clinician visits may focus predominantly on building a relationship with a patient to understand their motivations, interests, and facilitators and barriers to behavior change. The clinician can then use this information to relay relevant knowledge, initiate goal setting, and place relevant referrals. Follow-up visits can focus on reviewing goal progress, addressing barriers, correcting inaccurate information, providing motivation and brief education, and setting new goals [116]. Clinicians should provide patients with high-quality resources, such as those recommended in Supplementary Table S3 below, to review between visits, allowing for the most relationship-strengthening and high-yield conversations to happen during the visit. This both accommodates the busy clinician’s schedule and empowers the patient to take ownership of their health. The Sample Doctor-Patient Script in Supplementary Table S4 illustrates how nutritional assessment and counseling can be done in practice.

### Social Barriers: A Call to Action

While the primary goal of this review is to educate and empower individual clinicians to help individual patients implement nutrition-based interventions for the treatment of HTN, systemic change will be essential to allow for the large-scale adoption of effective, pragmatic, and affordable interventions. Clinicians should be educated and empowered to provide helpful cost-saving tips to improve financial accessibility, such as buying dry grains and legumes in bulk, frozen fruits and vegetables, and locally sourced food at community-based markets when available.

Community-level barriers to healthy eating include a high prevalence of businesses serving processed food, limited access to fresh and traditional foods, and urbanization [114]. Practices with ancillary support staff, such as social workers, can identify and leverage community assets and resources that help address these barriers. Partnerships with community organizations can promote urban farming initiatives and advocate for subsidies to improve physical and financial access to produce in food deserts. Clinicians can also advocate for medical school curricula and continuing medical education to develop more explicit content related to nutrition education and counseling so that tomorrow’s physicians are better equipped to educate and counsel patients about nutrition.

## Conclusion

Given its associated morbidity, mortality, and cost, it is imperative to utilize the most effective means possible to prevent and treat HTN. A preponderance of evidence across large-scale epidemiological studies, mechanistic studies, high-quality clinical trials, and meta-analyses supports adopting healthful plant-based dietary patterns to prevent the onset of HTN and improve outcomes among those already affected.

Clinicians can use evidence-based assessment tools and counseling techniques specifically designed to fit in with busy schedules. While individual and larger societal barriers to healthy plant-based diets exist, excellent resources are available to support and empower patients in their behavior change journey. However, addressing nutritional issues with patients is not solely the job of any one clinician. Collaboration between physicians, advanced practice providers, nurses, registered dieticians, social workers, health coaches, behavioral health specialists, and others will be crucial to providing patients with the expertise and support to enact sustained lifestyle changes.

Not all patients are interested in or capable of making significant dietary changes, and some patients will require pharmacologic intervention despite their best attempts. However, clinicians should still aim to address nutrition with their patients to fulfill their obligation to offer the most comprehensive and effective treatment plans, empowering patients to make more informed decisions about their care. The benefits of healthful plant-based nutrition are not limited to HTN, nor is diet the only important factor in addressing HTN. However, helping patients adopt a healthful plant-based diet is a powerful way to improve their health and blood pressure, one meal at a time.

### Supplementary Information

Below is the link to the electronic supplementary material.

Supplementary file1 (DOCX 26 KB)

## Data Availability

Data sharing is not applicable to this article as no new data were created or analyzed in this study.

## References

[1] GBD 2017 Risk Factor Collaborators. Global, regional, and national comparative risk assessment of 84 behavioural, environmental and occupational, and metabolic risks or clusters of risks for 195 countries and territories, 1990–2017: a systematic analysis for the Global Burden of Disease Study 2017. Lancet (Lond Engl). 2018;392:1923–94. 10.1016/S0140-6736(18)32225-6 PMC6227755 30496105

[2] Virani SS, Alonso A, Benjamin EJ, Bittencourt MS, Callaway CW, Carson AP, et al. Heart disease and stroke statistics—2020 update: a report from the American Heart Association. Circulation. 2020;141:e139–596. 10.1161/CIR.0000000000000757 31992061

[3] Rapsomaniki E, Timmis A, George J, Pujades-Rodriguez M, Shah AD, Denaxas S (2014). Blood pressure and incidence of twelve cardiovascular diseases: lifetime risks, healthy life-years lost, and age-specific associations in 1·25 million people. Lancet.

[4] Wu CY, Hu HY, Chou YJ, Huang N, Chou YC, Li CP. High blood pressure and all-cause and cardiovascular disease mortalities in community-dwelling older adults. Medicine (Baltimore). 2015;94:e2160. 10.1097/MD.0000000000002160 PMC5059018 26632749

[5] Aune D, Huang W, Nie J, Wang Y (2021). Hypertension and the risk of all-cause and cause-specific mortality: an outcome-wide association study of 67 causes of death in the National Health Interview Survey. BioMed Res Int.

[6] Ostchega Y, Fryar CD, Nwankwo T, Nguyen DT. Hypertension prevalence among adults aged 18 and over: United States, 2017–2018. NCHS Data Br. 2020;1–8. 32487290

[7] Mills KT, Stefanescu A, He J (2020). The global epidemiology of hypertension. Nat Rev Nephrol.

[8] Whelton PK, Carey RM, Aronow WS, Casey DE, Collins KJ, Dennison Himmelfarb C, et al. 2017 ACC/AHA/AAPA/ABC/ACPM/AGS/APhA/ASH/ASPC/NMA/PCNA Guideline for the prevention, detection, evaluation, and management of high blood pressure in adults: a report of the American College of Cardiology/American Heart Association task force on clinical practice guidelines. Hypertension. 2018;71:e13–115. 10.1161/HYP.0000000000000065 29133356

[9] Chobufo MD, Gayam V, Soluny J, Rahman EU, Enoru S, Foryoung JB, et al. Prevalence and control rates of hypertension in the USA: 2017–2018. Int J Cardiol Hypertens. 2020;6:100044. 10.1016/j.ijchy.2020.100044 PMC7803011 33447770

[10] Kirkland EB, Heincelman M, Bishu KG, Schumann SO, Schreiner A, Axon RN, et al. Trends in healthcare expenditures among US adults with hypertension: national estimates, 2003–2014. J Am Heart Assoc. 2018;7:e008731. 10.1161/JAHA.118.008731 PMC6015342 29848493

[11] Unger T, Borghi C, Charchar F, Khan NA, Poulter NR, Prabhakaran D (2020). 2020 International society of hypertension global hypertension practice guidelines. Hypertension.

[12] Williams B, Mancia G, Spiering W, Agabiti Rosei E, Azizi M, Burnier M (2018). 2018 ESC/ESH Guidelines for the management of arterial hypertension. Eur Heart J.

[13] Lichtenstein AH, Appel LJ, Vadiveloo M, Hu FB, Kris-Etherton PM, Rebholz CM (2021). 2021 dietary guidance to improve cardiovascular health: a scientific statement from the American Heart Association. Circulation.

[14] Bodai BI, Nakata TE, Wong WT, Clark DR, Lawenda S, Tsou C (2018). Lifestyle medicine: a brief review of its dramatic impact on health and survival. Perm J.

[15] Crowley J, Ball L, Hiddink GJ (2019). Nutrition in medical education: a systematic review. Lancet Planet Health.

[16] Aggarwal M, Devries S, Freeman AM, Ostfeld R, Gaggin H, Taub P (2018). The deficit of nutrition education of physicians. Am J Med.

[17] Hiddink GJ, Hautvast JG, van Woerkum CM, Fieren CJ, van’t Hof MA. Consumers’ expectations about nutrition guidance: the importance of primary care physicians. Am J Clin Nutr. 1997;65:1974S–9S. 10.1093/ajcn/65.6.1974S 9174506

[18] •• Joshi S, Ettinger L, Liebman SE. Plant-based diets and hypertension. Am J Lifestyle Med. 2020;14:397–405. **This review article outlines the evidence for plant-based diets in treating hypertension and highlights the mechanisms by which they work**. 10.1177/1559827619875411 PMC7692016 33281520

[19] Reddy KR. Cardiovascular disease and lifestyle medicine. J Fam Pract. 2022;71:S48–55. 10.12788/jfp.0251 35389845

[20] • Gardner CD, Vadiveloo MK, Petersen KS, Anderson CAM, Springfield S, Van Horn L, et al. Popular dietary patterns: alignment with American Heart Association 2021 dietary guidance: a scientific statement from the American Heart Association. Circulation. 2023;147:1715–30. **Created a tier system that highlights alignment of dietary patterns with AHA dietary guidelines, including facilitators and challenges to following them**. 10.1161/CIR.0000000000001146 37128940

[21] Tomé-Carneiro J, Visioli F (2023). Plant-based diets reduce blood pressure: a systematic review of recent evidence. Curr Hypertens Rep.

[22] Gibbs J, Gaskin E, Ji C, Miller MA, Cappuccio FP (2021). The effect of plant-based dietary patterns on blood pressure: a systematic review and meta-analysis of controlled intervention trials. J Hypertens.

[23] Hauser ME, McMacken M, Lim A, Shetty P (2022). Nutrition-an evidence-based, practical approach to chronic disease prevention and treatment. J Fam Pract.

[24] Appel LJ, Moore TJ, Obarzanek E, Vollmer WM, Svetkey LP, Sacks FM, et al. A clinical trial of the effects of dietary patterns on blood pressure. DASH Collaborative Research Group. N Engl J Med. 1997;336:1117–24. 10.1056/NEJM199704173361601 9099655

[25] Arnett DK, Blumenthal RS, Albert MA, Buroker AB, Goldberger ZD, Hahn EJ, et al. 2019 ACC/AHA guideline on the primary prevention of cardiovascular disease: a report of the American College of Cardiology/American Heart Association Task Force on Clinical Practice Guidelines. Circulation. 2019;140:e596–646. 10.1161/CIR.0000000000000678 PMC7734661 30879355

[26] Svetkey LP, Sacks FM, Obarzanek E, Vollmer WM, Appel LJ, Lin PH, et al. The DASH diet, sodium intake and blood pressure trial (DASH-sodium): rationale and design. J Am Diet Assoc. 1999;99:S96–104. 10.1016/s0002-8223(99)00423-x 10450301

[27] Karanja NM, Obarzanek E, Lin PH, McCullough ML, Phillips KM, Swain JF, et al. Descriptive characteristics of the dietary patterns used in the dietary approaches to stop hypertension trial. J Am Diet Assoc. 1999;99:S19–27. 10.1016/s0002-8223(99)00412-5 10450290

[28] Theodoridis X, Chourdakis M, Chrysoula L, Chroni V, Tirodimos I, Dipla K (2023). Adherence to the DASH diet and risk of hypertension: a systematic review and meta-analysis. Nutrients.

[29] Filippou CD, Tsioufis CP, Thomopoulos CG, Mihas CC, Dimitriadis KS, Sotiropoulou LI (2020). Dietary approaches to stop hypertension (DASH) diet and blood pressure reduction in adults with and without hypertension: a systematic review and meta-analysis of randomized controlled trials. Adv Nutr.

[30] Juraschek SP, Miller ER, Weaver CM, Appel LJ (2017). Effects of sodium reduction and the DASH diet in relation to baseline blood pressure. J Am Coll Cardiol.

[31] Keys A, Menotti A, Karvonen MJ, Aravanis C, Blackburn H, Buzina R (1986). The diet and 15-year death rate in the seven countries study. Am J Epidemiol.

[32] Davis C, Bryan J, Hodgson J, Murphy K (2015). Definition of the Mediterranean diet: a literature review. Nutrients.

[33] Filippou CD, Thomopoulos CG, Kouremeti MM, Sotiropoulou LI, Nihoyannopoulos PI, Tousoulis DM (2021). Mediterranean diet and blood pressure reduction in adults with and without hypertension: a systematic review and meta-analysis of randomized controlled trials. Clin Nutr.

[34] Nissensohn M, Román-Viñas B, Sánchez-Villegas A, Piscopo S, Serra-Majem L. The effect of the Mediterranean diet on hypertension: a systematic review and meta-analysis. J Nutr Educ Behav. 2016;48:42–53.e1. 10.1016/j.jneb.2015.08.023 26483006

[35] Trichopoulou A, Bamia C, Trichopoulos D. Anatomy of health effects of Mediterranean diet: Greek EPIC prospective cohort study. BMJ. 2009;338:b2337. 10.1136/bmj.b2337 PMC3272659 19549997

[36] Griswold MG, Fullman N, Hawley C, Arian N, Zimsen SRM, Tymeson HD (2018). Alcohol use and burden for 195 countries and territories, 1990–2016: a systematic analysis for the Global Burden of Disease Study 2016. Lancet.

[37] Appleby PN, Davey GK, Key TJ (2002). Hypertension and blood pressure among meat eaters, fish eaters, vegetarians and vegans in EPIC-Oxford. Public Health Nutr.

[38] Pettersen BJ, Anousheh R, Fan J, Jaceldo-Siegl K, Fraser GE (2012). Vegetarian diets and blood pressure among white subjects: results from the Adventist Health Study-2 (AHS-2). Public Health Nutr.

[39] Fraser G, Katuli S, Anousheh R, Knutsen S, Herring P, Fan J (2015). Vegetarian diets and cardiovascular risk factors in black members of the Adventist Health Study-2. Public Health Nutr.

[40] Yokoyama Y, Nishimura K, Barnard ND, Takegami M, Watanabe M, Sekikawa A (2014). Vegetarian diets and blood pressure: a meta-analysis. JAMA Intern Med.

[41] Lopez PD, Cativo EH, Atlas SA, Rosendorff C. The effect of vegan diets on blood pressure in adults: a meta-analysis of randomized controlled trials. Am J Med. 2019;132:875–83.e7. 10.1016/j.amjmed.2019.01.044 30851264

[42] Aljuraiban G, Chan Q, Gibson R, Stamler J, Daviglus ML, Dyer AR (2020). Association between plant-based diets and blood pressure in the INTERMAP study. BMJ Nutr Prev Health.

[43] Ostfeld RJ. Definition of a plant-based diet and overview of this special issue. J Geriatr Cardiol. 2017;14:315. 10.11909/j.issn.1671-5411.2017.05.008 PMC5466934 28630607

[44] Elsahoryi NA, Neville CE, Patterson CC, Linden GJ, Moitry M, Biasch K (2021). Association between overall fruit and vegetable intake, and fruit and vegetable sub-types and blood pressure: the PRIME study (prospective epidemiological study of myocardial infarction). Br J Nutr.

[45] Hartley M, Fyfe CL, Wareham NJ, Khaw K-T, Johnstone AM, Myint PK (2022). Association between legume consumption and risk of hypertension in the European prospective investigation into cancer and nutrition (EPIC)-Norfolk cohort. Nutrients.

[46] Kirwan JP, Malin SK, Scelsi AR, Kullman EL, Navaneethan SD, Pagadala MR (2016). A whole-grain diet reduces cardiovascular risk factors in overweight and obese adults: a randomized controlled trial 123. J Nutr.

[47] Bae YJ, Kim MH, Choi MK (2022). Dietary mineral intake from nuts and its relationship to hypertension among Korean adults. Biol Trace Elem Res.

[48] Petersen KS, Davis KM, Rogers CJ, Proctor DN, West SG, Kris-Etherton PM (2021). Herbs and spices at a relatively high culinary dosage improves 24-hour ambulatory blood pressure in adults at risk of cardiometabolic diseases: a randomized, crossover, controlled-feeding study. Am J Clin Nutr.

[49] Linderman GC, Lu J, Lu Y, Sun X, Xu W, Nasir K, et al. Association of Body Mass Index With Blood Pressure Among 1.7 Million Chinese Adults. JAMA Netw Open. 2018;1:e181271. 10.1001/jamanetworkopen.2018.1271 PMC6324286 30646115

[50] Aucott L, Poobalan A, Smith WCS, Avenell A, Jung R, Broom J (2005). Effects of weight loss in overweight/obese individuals and long-term hypertension outcomes. Hypertension..

[51] Wright N, Wilson L, Smith M, Duncan B, McHugh P (2017). The BROAD study: a randomised controlled trial using a whole food plant-based diet in the community for obesity, ischaemic heart disease or diabetes. Nutr Diabetes.

[52] Grillo A, Salvi L, Coruzzi P, Salvi P, Parati G (2019). Sodium intake and hypertension. Nutrients.

[53] Geleijnse JM, Kok FJ, Grobbee DE (2004). Impact of dietary and lifestyle factors on the prevalence of hypertension in Western populations. Eur J Public Health.

[54] Harnack LJ, Cogswell ME, Shikany JM, Gardner CD, Gillespie C, Loria CM (2017). Sources of sodium in US adults from 3 geographic regions. Circulation.

[55] Huang L, Trieu K, Yoshimura S, Neal B, Woodward M, Campbell NRC, et al. Effect of dose and duration of reduction in dietary sodium on blood pressure levels: systematic review and meta-analysis of randomised trials. BMJ. 2020;368:m315. 10.1136/bmj.m315 PMC7190039 32094151

[56] Whelton PK, He J, Appel LJ, Cutler JA, Havas S, Kotchen TA (2002). Primary prevention of hypertension: clinical and public health advisory from The National High Blood Pressure Education Program. JAMA.

[57] Adrogué HJ, Madias NE (2014). The impact of sodium and potassium on hypertension risk. Semin Nephrol.

[58] Murillo-de-Ozores AR, Gamba G, Castañeda-Bueno M (2019). Molecular mechanisms for the regulation of blood pressure by potassium. Curr Top Membr.

[59] Levings JL, Gunn JP (2014). The imbalance of sodium and potassium intake: implications for dietetic practice. J Acad Nutr Diet.

[60] Perez V, Chang ET (2014). Sodium-to-potassium ratio and blood pressure, hypertension, and related factors. Adv Nutr.

[61] Iwahori T, Miura K, Ueshima H, Tanaka-Mizuno S, Chan Q, Arima H (2019). Urinary sodium-to-potassium ratio and intake of sodium and potassium among men and women from multiethnic general populations: the INTERSALT study. Hypertens Res.

[62] Centers for Disease Control and Prevention (CDC). Usual sodium intakes compared with current dietary guidelines---United States, 2005–2008. MMWR Morb Mortal Wkly Rep. 2011;60:1413–7. 22012113

[63] Hoy MK, Goldman JD, Moshfegh A. Potassium intake of the U.S. population: what we eat in America, NHANES 2017–2018. FSRG Diet Data Briefs [Internet]. Beltsville (MD): United States Department of Agriculture (USDA); 2010[cited 2023 Sep 12]. Available from: http://www.ncbi.nlm.nih.gov/books/NBK587683/. 36630549

[64] Gröber U, Schmidt J, Kisters K (2015). Magnesium in prevention and therapy. Nutrients.

[65] Houston M (2011). The role of magnesium in hypertension and cardiovascular disease. J Clin Hypertens Greenwich Conn.

[66] Villa-Etchegoyen C, Lombarte M, Matamoros N, Belizán JM, Cormick G (2019). Mechanisms involved in the relationship between low calcium intake and high blood pressure. Nutrients.

[67] Zheng MH, Li FXZ, Xu F, Lin X, Wang Y, Xu QS, et al. The interplay between the renin-angiotensin-aldosterone system and parathyroid hormone. Front Endocrinol. 2020;11:539. 10.3389/fendo.2020.00539 PMC7468498 32973674

[68] Jayedi A, Zargar MS (2019). Dietary calcium intake and hypertension risk: a dose-response meta-analysis of prospective cohort studies. Eur J Clin Nutr.

[69] Cormick G, Ciapponi A, Cafferata ML, Cormick MS, Belizán JM. Calcium supplementation for prevention of primary hypertension. Cochrane Database Syst Rev. 2022;1:CD010037. 10.1002/14651858.CD010037.pub4 PMC8748265 35014026

[70] Quagliani D, Felt-Gunderson P (2016). Closing America’s fiber intake gap. Am J Lifestyle Med.

[71] Anderson JW, Baird P, Davis RH, Ferreri S, Knudtson M, Koraym A (2009). Health benefits of dietary fiber. Nutr Rev.

[72] Streppel MT, Arends LR, van’t Veer P, Grobbee DE, Geleijnse JM. Dietary fiber and blood pressure: a meta-analysis of randomized placebo-controlled trials. Arch Intern Med. 2005;165:150–6. 10.1001/archinte.165.2.150 15668359

[73] Sidhu SRK, Kok CW, Kunasegaran T, Ramadas A (2023). Effect of plant-based diets on gut microbiota: a systematic review of interventional studies. Nutrients.

[74] Yang Z, Wang Q, Liu Y, Wang L, Ge Z, Li Z, et al. Gut microbiota and hypertension: association, mechanisms and treatment. Clin Exp Hypertens NYN. 1993;2023(45):2195135. 10.1080/10641963.2023.2195135 36994745

[75] Xu JW, Ikeda K, Yamori Y (1979). Upregulation of endothelial nitric oxide synthase by cyanidin-3-glucoside, a typical anthocyanin pigment. Hypertens Dallas Tex.

[76] Achike FI, Kwan C-Y (2003). Nitric oxide, human diseases and the herbal products that affect the nitric oxide signalling pathway. Clin Exp Pharmacol Physiol.

[77] Bahadoran Z, Mirmiran P, Kabir A, Azizi F, Ghasemi A (2017). The nitrate-independent blood pressure-lowering effect of beetroot juice: a systematic review and meta-analysis. Adv Nutr Bethesda Md.

[78] Bonilla Ocampo DA, Paipilla AF, Marín E, Vargas-Molina S, Petro JL, Pérez-Idárraga A. Dietary nitrate from beetroot juice for hypertension: a systematic review. Biomolecules. 2018;8. 10.3390/biom8040134 PMC6316347 30400267

[79] Benjamim CJR, Porto AA, Valenti VE, Sobrinho AC, Garner DM, Gualano B, et al. Nitrate derived from beetroot juice lowers blood pressure in patients with arterial hypertension: a systematic review and meta-analysis. Front Nutr. 2022;9:823039. 10.3389/fnut.2022.823039 PMC8965354 35369064

[80] Mitchell T, Kumar P, Reddy T, Wood KD, Knight J, Assimos DG (2019). Dietary oxalate and kidney stone formation. Am J Physiol - Ren Physiol.

[81] Shakil MH, Trisha AT, Rahman M, Talukdar S, Kobun R, Huda N (2022). Nitrites in cured meats, health risk issues, alternatives to nitrites: a review. Foods.

[82] Kotopoulou S, Zampelas A, Magriplis E (2023). Nitrite and nitrate intake from processed meat is associated with elevated diastolic blood pressure (DBP). Clin Nutr Edinb Scotl.

[83] Bahrampour N, Mirzababaei A, Hosseininasab D, Abaj F, Clark CCT, Mirzaei K (2023). High intake of dietary phytochemical index may be related to reducing risk of diabetic nephropathy: a case-control study. BMC Nutr.

[84] Jasemi SV, Khazaei H, Aneva IY, Farzaei MH, Echeverría J (2020). Medicinal plants and phytochemicals for the treatment of pulmonary hypertension. Front Pharmacol.

[85] Verma T, Sinha M, Bansal N, Yadav SR, Shah K, Chauhan NS (2021). Plants used as antihypertensive. Nat Prod Bioprospecting.

[86] Steffen LM, Kroenke CH, Yu X, Pereira MA, Slattery ML, Van Horn L, et al. Associations of plant food, dairy product, and meat intakes with 15-y incidence of elevated blood pressure in young black and white adults: the Coronary Artery Risk Development in Young Adults (CARDIA) study. Am J Clin Nutr. 2005;82:1169–77; quiz 1363–4. 10.1093/ajcn/82.6.1169 16332648

[87] Borgi L, Curhan GC, Willett WC, Hu FB, Satija A, Forman JP (2015). Long-term intake of animal flesh and risk of developing hypertension in three prospective cohort studies. J Hypertens.

[88] Schwingshackl L, Schwedhelm C, Hoffmann G, Knüppel S, Iqbal K, Andriolo V (2017). Food groups and risk of hypertension: a systematic review and dose-response meta-analysis of prospective studies. Adv Nutr Bethesda Md.

[89] Gou R, Gou Y, Qin J, Luo T, Gou Q, He K (2022). Association of dietary intake of saturated fatty acids with hypertension: 1999–2018 National Health and Nutrition Examination Survey. Front Nutr.

[90] MacDonald CJ, Madkia A-L, Mounier-Vehier C, Severi G, Boutron-Ruault M-C (2023). Associations between saturated fat intake and other dietary macronutrients and incident hypertension in a prospective study of French women. Eur J Nutr.

[91] Vasdev S, Gill V, Singal P (2007). Role of advanced glycation end products in hypertension and atherosclerosis: therapeutic implications. Cell Biochem Biophys.

[92] Mutengo KH, Masenga SK, Mweemba A, Mutale W, Kirabo A (2023). Gut microbiota dependant trimethylamine N-oxide and hypertension. Front Physiol.

[93] • Hull SC, Charles J, Caplan AL. Are we what we eat? The moral imperative of the medical profession to promote plant-based nutrition. Am J Cardiol. 2023;188:15–21. **This editorial addresses common misperceptions about plant-based nutrition and meat consumption and describes the unhealthful components of animal foods**. 10.1016/j.amjcard.2022.10.006 36446227

[94] Satija A, Hu FB (2018). Plant-based diets and cardiovascular health. Trends Cardiovasc Med.

[95] ESHA’s Food Processor^®^ Nutrition Analysis [Internet]. Salem, OR: Food Processor SQL Inc; 2008[cited 2023 Sep 7]. Available from https://esha.com/products/food-processor/.

[96] US Department of Agriculture Agricultural Research Service. FoodData Central [Internet]. 2019. Available from fdc.nal.usda.gov.

[97] Paterick TJ, Carson GV, Allen MC, Paterick TE (2008). Medical informed consent: general considerations for physicians. Mayo Clin Proc.

[98] Kahan S, Manson JE (2017). Nutrition counseling in clinical practice: how clinicians can do better. JAMA.

[99] Vadiveloo M, Lichtenstein AH, Anderson C, Aspry K, Foraker R, Griggs S, et al. Rapid diet assessment screening tools for cardiovascular disease risk reduction across healthcare settings: a scientific statement from the American Heart Association. Circ Cardiovasc Qual Outcomes. 2020;13:e000094. 10.1161/HCQ.0000000000000094 32762254

[100] Powell HS, Greenberg DL. Screening for unhealthy diet and exercise habits: the electronic health record and a healthier population. Prev Med Rep. 2019;14:100816. 10.1016/j.pmedr.2019.01.020 PMC6377403 30815334

[101] Paxton AE, Strycker LA, Toobert DJ, Ammerman AS, Glasgow RE (2011). Starting the conversation: performance of a brief dietary assessment and intervention tool for health professionals. Am J Prev Med.

[102] Papadaki A, Johnson L, Toumpakari Z, England C, Rai M, Toms S (2018). Validation of the English version of the 14-item Mediterranean diet adherence screener of the PREDIMED study, in people at high cardiovascular risk in the UK. Nutrients.

[103] Naska A, Lagiou A, Lagiou P. Dietary assessment methods in epidemiological research: current state of the art and future prospects. F1000Res. 2017;6:926. 10.12688/f1000research.10703.1 PMC5482335 28690835

[104] Matthews JA, Moore M, Collings C. A coach approach to facilitating behavior change. J Fam Pract. 2022;71:eS93–9. 10.12788/jfp.0246 35389854

[105] Vallis M, Piccinini-Vallis H, Sharma AM, Freedhoff Y (2013). Modified 5 As. Can Fam Physician.

[106] Gutnick D, Reims K, Davis C, Gainforth H, Jay M, Cole S (2014). Brief action planning to facilitate behavior change and support patient self-management. JCOM.

[107] Miller WR, Rollnick S. Motivational interviewing: helping people change. Guilford Press; 2012.

[108] Martins RK, McNeil DW (2009). Review of motivational interviewing in promoting health behaviors. Clin Psychol Rev.

[109] Thompson DR, Chair SY, Chan SW, Astin F, Davidson PM, Ski CF (2011). Motivational interviewing: a useful approach to improving cardiovascular health?. J Clin Nurs.

[110] Huang X, Xu N, Wang Y, Sun Y, Guo A. The effects of motivational interviewing on hypertension management: a systematic review and meta-analysis. Patient Educ Couns. 2023;112:107760. 10.1016/j.pec.2023.107760 37075650

[111] Cole SA, Sannidhi D, Jadotte YT, Rozanski A (2023). Using motivational interviewing and brief action planning for adopting and maintaining positive health behaviors. Prog Cardiovasc Dis.

[112] Bailey RR (2017). Goal setting and action planning for health behavior change. Am J Lifestyle Med.

[113] Wetherill M, Whelan L, Do L, Schumann S-A, Davis G, Carter V (2019). Nutrition prescriptions made simple. J Okla State Med Assoc.

[114] Fenta ET, Tiruneh MG, Anagaw TF (2023). Exploring enablers and barriers of healthy dietary behavior based on the socio-ecological model, a qualitative systematic review. Nutr Diet Suppl.

[115] Robinson T (2008). Applying the socio-ecological model to improving fruit and vegetable intake among low-income African Americans. J Community Health.

[116] Karlsen MC, Pollard KJ. Strategies for practitioners to support patients in plant-based eating. J Geriatr Cardiol 2017;14:338–41. 10.11909/j.issn.1671-5411.2017.05.006 PMC5466940 28630613

[117] Bharati R, Kovach KA, Bonnet JP, Sayess P, Polk E, Harvey K (2023). Incorporating lifestyle medicine into primary care practice: perceptions and practices of family physicians. Am J Lifestyle Med.

[118] Adamski M, Gibson S, Leech M, Truby H (2018). Are doctors nutritionists? What is the role of doctors in providing nutrition advice?. Nutr Bull.

[119] Hickson M, Child J, Collinson A. A case study of the impact of a dietitian in the multi-disciplinary team within primary care: a service evaluation. J Hum Nutr Diet Off J Br Diet Assoc. 2023. 10.1111/jhn.13217 37526210

[120] Sforzo GA, Kaye MP, Todorova I, Harenberg S, Costello K, Cobus-Kuo L (2018). Compendium of the health and wellness coaching literature. Am J Lifestyle Med.

[121] Sforzo GA, Kaye MP, Harenberg S, Costello K, Cobus-Kuo L, Rauff E (2020). Compendium of health and wellness coaching: 2019 addendum. Am J Lifestyle Med.

[122] Burgess E, Hassmén P, Welvaert M, Pumpa KL (2017). Behavioural treatment strategies improve adherence to lifestyle intervention programmes in adults with obesity: a systematic review and meta-analysis. Clin Obes.

