# Supplementary material for: Planting the Seed for Blood Pressure Control: The Role of Plant-Based Nutrition in the Management of Hypertension
Source: Curr Cardiol Rep. 2024 Mar 25;26(3):121–34. doi: 10.1007/s11886-023-02008-z (PMC10990999; doi:10.1007/s11886-023-02008-z)
Supplement: Supplementary file 1 — Supplementary file1 (DOCX 26 KB) [file 11886_2023_2008_MOESM1_ESM.docx]

**Supplementary Tables**

**Table S1: SMART Goals for Nutrition Prescriptions**

| **S**pecific | Describe what foods are being prescribed. | “Eat ½ cup of **broccoli** each dinner” |
| --- | --- | --- |
| **M**easurable | Describe the quantity that is being prescribed. | “Eat **1 cup** of cooked spinach at lunch” |
| **A**chievable/attainable | Describe how the goals will be achieved.   - Who will do the shopping? - How often will produce be purchased? - How can meal prepping be done? | Rate confidence on scale of 0-10 (10 = 100% confidence)  “What will make it easier for you to follow this plan?”  “Will it be easier to cut and freeze/refrigerate the vegetables ready-to-use to make it easier for you to cook your meals during the work week?” |
| **R**ealistic/relevant | Set realistic & relevant goals tailored for the patient. Start small. | Check-in regularly with the patient to determine what is working and challenges encountered. Course-correct based on patient feedback, as needed |
| **T**ime-bound | Describe how often and the duration that the nutrition prescription plan will be followed.  Start with a lower frequency and build it up gradually based on patient’s feedback to promote adherence and self-efficacy. | You may wish to prescribe the plan for a period of 3- 6 months.  Note frequency in a week (e.g. 3 times a week)  Note days when the patient will and won’t follow plan to allow for flexibility. |

**Table S2: Developing a SMART nutrition prescription**

| **5 *A*s Technique** | **Brief Action Planning (BAP) Step** | **Clinician’s Role** | **SMART nutrition prescription for HTN** |
| --- | --- | --- | --- |
| Ask | Elicit a behavior goal | Assess the patient’s readiness and explore internal motivation  Seek permission to propose a collaboratively developed plan. | Components of a nutrition prescription:   - T – Type of food (also indicate how it is prepared) - A – Amount of food (cups/tsp/tbsp) - F – Frequency of consumption (during a week, and period of 3-6 months)     **Example positive prescriptions (to increase healthful foods):**  **Fiber:**   - *“Eat ½ cup of steamed broccoli or spinach for lunch every day for 6 months”* - *“Eat ½ cup cooked lentils for dinner 3 times a week for a period of 3 months”*     **Magnesium & Potassium:**   - *“Eat ¼ cup of cooked tofu for lunch 2-3 times a week for 3 months”* - *“Eat ½ banana for with 2 squares of dark chocolate for dessert”*     **Example negative prescriptions (to decrease unhealthful foods):**  **Sodium:**   - *“Consume no more than ¾ tsp (1500mg) of salt daily, 5 times per week for 1 month”* - *“Use lemon juice and other herbs to replace half of the salt used while cooking twice weekly for 6 weeks*”   **Saturated fat**   - *“Replace butter or margarine with unsaturated plant-based oils, such as olive or avocado oil every other time you cook for 2 weeks*     **Processed foods:**   - *“Limit chips, deli meats, and processed plant-based “meats” to one weekend per month for 3 months”* - *“Limit take-out and restaurant meals to twice weekly for 2 months”* |
| Assess |  | Inquire about the patient’s regular dietary habits  Set goals based on what is practical. |  |
| Advise |  | Develop SMART nutritional targets for the patient.  To start, focus on adding healthy foods to their regular diet and replacing them with healthier options. |  |
| Agree | Elicit a commitment  Assess confidence to implement the plan | Assess understanding of goals and the nutrition prescription. |  |
| Assist | Arrange accountability | Counsel, and provide self-help resources and materials; arrange follow-up to assess progress. |  |

| **Table S3: Strategies to Address Patient Barriers to Adopting Plant-Based Diets** | | |
| --- | --- | --- |
| **Scenario** | **Patient Perspective** | **Recommended Resources** |
| Patient lacks sufficient knowledge | “I’m not sure what it means to follow a healthy diet. What should I eat?” | - [ACLM Food is Medicine Jumpstart](https://lifestylemedicine.org/wp-content/uploads/2022/07/ACLM-Food-As-Medicine-Jumpstart-8.5x11.pdf) - [Plantrician Project Plant-Based Nutrition Quick Start Guide](https://plantricianproject.org/quickstartguide) - [Kaiser Permanente Plant-Based Diet Guide](https://healthy.kaiserpermanente.org/content/dam/kporg/final/documents/health-education-materials/instructions/plant-based-diet-hi-en.pdf) |
| Patient has difficulty initiating behavior | “I know I should eat healthier, but I don’t have the skills to cook or plan my meals” | Cookbooks   - [Bravo! Health Promoting Meals from the TrueNorth Health Kitchen](https://www.bravopb.com/cookbooks/) - [Forks over Knives – The Cookbook](https://shop.forksoverknives.com/collections/books/products/forks-over-knives-the-cookbook)   Culinary Instruction   - [Roxube Plant-Based Cooking Introduction](https://rouxbe.com/plant-based-introduction/) - [Forks Over Knives Plant-Based Cooking Essentials](https://www.forksoverknives.com/cooking-course/?gad=1&gclid=CjwKCAjwu4WoBhBkEiwAojNdXte2el5boaYfVwPhu10JmKTMzniA7fkmUcpz9pfG2jr37JvvWBbJYhoChuYQAvD_BwE)   Referrals   - Find plant-based clinicians using the [ACLM](https://www.lifestylemedpros.org/home) and [Plantrician Project](https://www.plantrician.org/) Directories - Refer to Therapeutic Lifestyle Change programs, like [Pivio](https://piviohealth.com/) |
| Patient lacks motivation | “I know I don’t eat as well as I should, I just don’t feel inspired to make changes” | Goal Setting   - Encourage SMART goal setting (See Table S1)   Social Support   - Encourage joining/starting a [Plant-Based Nutrition Support Group](https://www.pbnsg.org/)or [PlantPure Communities Pod](https://plantpurecommunities.org/pod-network/)   Modeling/Success Stories   - [Forks Over Knives Success Stories](about:blank) |

**Table S4: Sample Doctor-Patient Script**

| D = Doctor  P = Patient |
| --- |
| *D: Thanks for coming in today Mr. Smith, we reviewed your blood pressure log and your numbers are still higher than we’d like them to be. To get to know you better, I want to know your general goals with your health and blood pressure.*  *P: Well, I want my blood pressure to be controlled.*  *D: And why is that?*  *P: Well, I don’t want to have complications like strokes and heart attacks. I want to be healthy.*  *D: What motivates you to be healthy?*  *P: I want to be around for my family.*  *D: Say more. Why is that important to you?*  *P: Well, my dad had high blood pressure and never took care of himself. He had a stroke that left him paralyzed, and my mom, my siblings, and I had to help care for him. He had to take all of these pills every day and couldn’t do the things he loved. He died when I was 14, and I’m still not over it. I don’t want to have to be dependent on my kids or for my kids to grow up without a dad. Being on these pills makes me scared that I’m going to end up like him.*  *D: Thank you so much for sharing that. Your family is really important to you, and you want to do everything you can to avoid having what happened to your dad happen to you.*  *P: Yeah, doc, what can I do besides just keep taking these pills?*  *D: Well, I’d like to discuss some other tools you can use to help manage your blood pressure.*  *P: Tools, like what?*  *D: Well, we know from almost a century of research that eating a healthy diet can not only prevent but even treat or reverse high blood pressure. Optimizing diet can even help certain patients get off of some or all of their medications. Would you be open to discussing your diet?*  *P: Sure, well, I eat pretty healthy. What do you want to know?*  *D: Well, I want to learn more about what you eat on a day-to-day basis, then talk to you about the components of really healthy diets, then if you’re interested, we can set a goal, and I can give you some resources.*  *P: Ok, sounds good.*  *D: Great, so tell me in as much detail as possible everything you ate and drank yesterday from when you woke up to when you went to bed.*  *P: Well, I woke up around 7 AM and had some eggs and toast for breakfast with a coffee. Then for lunch, I had a turkey sandwich with some salad on the side. I had another cup of coffee around 3 PM, and then for dinner, I had grilled chicken with string beans and some rice.*  *D: Ok, thanks for sharing all of that! So tell me in more detail about your breakfast; how many eggs did you have and how did you prepare them?*  *P: I had two eggs over-easy fried in butter.*  *D: Ok, and what else did you have with the eggs?*  *P: I had two strips of bacon and toast.*  *D: What kind of toast and how did you make it?*  *P: It was wheat bread and I had some butter and jam on it.*  *D: Got it! And how do you take your coffee?*  *P: I put in some half and half and 3 sugars.*  *D: Ok, and did you eat anything between breakfast and the sandwich for lunch?*  *P: Oh, yeah, someone brought in donuts, so I had a jelly donut around 10:30 AM.*  *D: Anything else before lunch?*  *P: Nope!*  *D: Ok, tell me what was on your sandwich in as much detail as you can.*  *P: I had 4 slices of deli turkey with 2 slices of swiss cheese, lettuce, tomato, and mayo on whole wheat bread.*  *D: And what was in the salad?*  *P: Lettuce, tomato, cucumber, onions and ranch dressing.*  *D: Did you take your afternoon coffee the same as your morning coffee?*  *P: Yes, half and half and 3 sugars.*  *D: Ok, anything else between lunch and dinner?*  *P: No, just the coffee.*  *D: Alright, and for dinner, how much of your plate was chicken, string beans, and rice in relation to the size of your palm?*  *P: The chicken was about twice the size of my palm, and the string beans and rice were maybe half a palm each. I’d say half the plate was chicken, one quarter was rice and one quarter were string beans.*  *D: And what type of rice did you have?*  *P: White rice, made with butter and a seasoning packet.*  *D: Did you have any drinks with dinner or eat anything for dessert?*  *P: I had a glass of red wine with dinner and oh yeah, a quarter of a chocolate bar and some strawberries for dessert.*  *D: Was there any other food or drink we missed, even if it was only a few small bites?*  *P: I had a few nibbles of cheddar cheese from the fridge when I got home, probably about half a slice.*  *D: Thanks for going through all of that with me! On a scale from 0-10 where 10 is the healthiest diet imaginable, how would you rate your diet and why?*  *P: I’d say a 7 or 8*  *D: Great, and why a 7 or 8 and not a 5 or 6?*  *P: Well, I eat my vegetables, I don’t eat fast food, and I don’t drink any soda!*  *D: That makes sense! And why not a 9 or 10?*  *P: Well, I probably shouldn’t have had the chocolate after dinner, and I should really cut down on the carbs like the rice.*  *D: I think you’re doing a lot well too! You’re eating 3 servings of vegetables every day, you’re avoiding sugar-sweetened beverages, and you’re mostly eating at home! I think there are some things we can improve on if you’d like, too. Do you mind if I share some information about what a diet with a score of 10 might look like?*  *P: Sure!*  *D: So what I recommend, based on a lot of the scientific evidence, is something called a whole food, plant-based diet. Have you heard of that before?*  *P: You mean like those fake meats and cheeses?*  *D: Not exactly. I like to break it into two parts, the plant-based part, and the whole food part. “Plant-Based” means eating mostly food from plants instead of animals. This means eating more vegetables, fruits, whole grains, and beans, with some nuts, seeds, and avocados. It also means avoiding or eliminating red meat, pork, poultry, fish, eggs, and dairy. This is because animal-based foods tend to cause disease, while plant-based foods tend to help your body heal. “Whole-Food” means eating food in its most whole, natural form and avoiding processed foods. Think of it as food your great-great-grandparents would recognize as real food. This means that all the fiber and nutrients are kept in, while the added sugar, salt, oils, and chemicals are kept out. We like to tell people to eat apples, instead of applesauce or apple pie. You should look for foods that have as few ingredients as possible, and ideally, they don’t even have a nutrition label, because they’re just one ingredient, like broccoli, black beans, or brown rice! It also means processed “vegan” foods like fake meats and cheese are not part of this diet.*  *I know that was a lot of information; what are your thoughts?*  *P: Well, doc, I don’t think I’m ready to give up meat. I could never be a vegan! Is that the only way I can be healthy?*  *D: Not at all. My job is to help you figure out your goals and then determine a plan to help you meet them. Not everyone needs to be at a 10! Diet is a spectrum, and any changes we make towards increasing the amount of minimally processed plant-based foods and decreasing the amount of animal-based and processed foods, the more we improve your health!*  *P: Ok, that sounds fair. I really do want to get my blood pressure under better control. So what should I do?*  *D: Some people like to make big changes all at once and others like to make slow changes one step at a time. What usually works for you?*  *P: I like to go slow and steady otherwise, I just stop after a few days and go back to what I was doing before.*  *D: That’s helpful to know! Are you interested in making any changes to your diet?*  *P: Well, I don’t want to give up my meat, but I could eat less red meat. I make a lot of chili and usually put ground beef in it. You kept mentioning how healthy beans are. Maybe I could use those instead?*  *D: That sounds like a great idea! Is that the sort of goal you’d like to set?*  *P: Yeah, that sounds good!*  *D: Ok, let’s try to get specific. Tell me your plan in as much detail as possible.*  *P: Ok, well, I usually make a big batch of chili on Sunday and eat it three times a week. Next week, I could use kidney beans instead of ground beef in the recipe, so I’d cut out beef for three meals.*  *D: Great! How confident are you on a scale of 0-10, where 10 is 100% confidence that you can meet this goal?*  *P: I’d say an 8. My wife and kids would probably be fine with that since they’re always telling me to cut back on red meat too.*  *D: You have a great support system to help you with this goal. That’s excellent!*  *P: Yeah! I’m actually excited! I’m still not totally sure about what this plant-based diet is. How can I learn more about it?*  *D: I’ll send you some more resources on the patient portal with links to some great informational videos and recipes! When people are making significant changes like you are, it can be helpful to have frequent check-ins. Work with dieticians and health coaches can help you plan your diet to get all the necessary nutrients, work through issues, and set and refine your goals. Would you like to work with our dietician or health coach between now and our next visit?*  *P: Sure, whatever will help!*  *D: Great, I’ll send referrals over right now, and they’ll call you to check in in 2 weeks. If you’re able to make these changes, I think you’ll really improve your blood pressure and your overall health*  *P: Thanks, doc!* |
